# Project Masihambisane: a cluster randomised controlled trial with peer mentors to improve outcomes for pregnant mothers living with HIV

**DOI:** 10.1186/1745-6215-12-2

**Published:** 2011-01-04

**Authors:** Mary-Jane Rotheram-Borus, Linda Richter, Heidi Van Rooyen, Alastair van Heerden, Mark Tomlinson, Alan Stein, Tamsen Rochat, Julia de Kadt, Nonhle Mtungwa, Lungile Mkhize, Lindo Ndlovu, Lungile Ntombela, W Scott Comulada, Katherine A Desmond, Erin Greco

**Affiliations:** 1 Global Center for Children and Families, University of California at Los Angeles, California, USA; 2 Child, Youth, Family and Social Development, Human Sciences Research Council, Pretoria, South Africa; 3 School of Psychology, University of KwaZulu-Natal, Durban, South Africa; 4 Department of Psychiatry, Oxford University, Oxford, UK; 5 Africa Centre for Health and Population Studies, Mtubatuba, Durban, South Africa; 6 Department of Psychology, University of Stellenbosch, Stellenbosch, South Africa

## Abstract

**Background:**

Pregnant women living with HIV (WLH) face daily challenges maintaining their own and their babies' health and mental health. Standard Prevention of Maternal to Child Transmission (PMTCT) programs are not designed to address these challenges.

**Methods/Design:**

As part of a cluster randomized controlled trial, WLH are invited to attend four antenatal and four postnatal small group sessions led by a peer WLH (a Peer Mentor). The WLH and their babies are assessed during pregnancy and at one week, six months, and twelve months post-birth. Mobile phones are used to collect routine information, complete questionnaires and remain in contact with participants over time. Pregnant WLH (N = 1200) are randomly assigned by clinic (N = 8 clinics) to an intervention program, called *Masihambisane *(n = 4 clinics, n = 600 WLH) or a standard care PMTCT control condition (n = 4 clinics; n = 600 WLH).

**Discussion:**

Data collection with cellular phones are innovative and effective in low-resource settings. Standard PMTCT programs are not designed to address the daily challenges faced by WLH; Peer Mentors may be useful in supporting WLH to cope with these challenges.

**Trial registration:**

ClinicalTrials.gov registration # NCT00972699

## Background

An estimated 1.4 million pregnant women are women living with HIV (WLH) in low and middle income countries (LMIC) [1]. South Africa has the highest total number of people living with HIV (5.7 million), of whom 3.2 million are women [1] and more than 200,000 are pregnant WLH in need of antiretrovirals [2]. The national HIV prevalence has stabilised around 11% [3], with the highest rates in the Kwa-Zulu-Natal and Gauteng Provinces. Up to 40-60% of pregnant women in Kwa-Zulu-Natal are HIV positive [4]. Antiretroviral (ARV) medications can reduce HIV transmission to less than 2% at childbirth and exclusive breastfeeding for six months also minimizes risk of transmission [5]. Therefore, effective programs to prevent mother-to-child transmission (PMTCT) for WLH in South Africa are urgently needed.

The goal of this cluster randomized controlled trial (RCT) is to evaluate a clinic-based strategy for increasing uptake of PMTCT and to improve maternal health behaviors over time. The *Masihambisane *program is leveraging the unique intervention opportunities created by pregnancy. Pregnancy requires mothers to adopt new routines and behaviors. HIV, alcohol and nutrition are linked to daily behaviors and habits, which are significantly easier to shift during life transitions compared to periods of stable living [6]. The birth of a child is a major life transition as relationships become reordered and there are changes in sleeping, eating, socializing, and working patterns. This creates the opportunity for "upstream" programming of new attitudes and behaviours [6].

WLH's experience of HIV-related stigma is often associated with poor antenatal and postnatal adherence to HIV-related regimens and healthy lifestyles [7,8]. Having a peer for support at the time of diagnoses who also introduces and supports health regimens (e.g., ARV medication adherence, exclusive breastfeeding) can be emotionally powerful [9]. There is a history of peer self-help interventions in LMIC that utilize peer advocates, including Alcoholics Anonymous [10], Widow-to-Widow [11], and community popular opinion leaders [12]. The mothers2mothers programme (m2m) is an HIV perinatal support program that initiated placing WLH as Peer Mentors in clinics beginning in 1990 http://www.m2m.org. The Masihambisane Project builds on the m2m programme by systematically training the Peer Mentor WLH to support PMTCT goals.

PMTCT programs typically focus on three biomedical interventions: maternal HIV testing; ARV provision in preparation for and during childbirth; and infant ARV medication adherence for six weeks until infant serostatus is established by PCR testing [5]. While ARV treatment is highly effective, only 21% of pregnant women in LMIC were tested for HIV in 2008, and fewer than half of those testing seropositive received ARV medication [1]. Beginning in 2001 [13], routine PMTCT has been available in South Africa. By 2007/08, 81% of pregnant women attending public antenatal clinics in South Africa had been tested. However, Kwa-Zulu-Natal has the lowest levels of testing at 71% [14]. Of the women tested for HIV, about a third do not receive their test results [3,15]. In 2007, 76*% *of pregnant WLH who did receive their test results received Nevirapine (NVP) during labour, while 57% of infants received NVP at birth [13,16].

Health service coverage for women and children in South Africa is good, as 92% of pregnant women have access to antenatal care [15]. Women generally begin antenatal care prior to the third trimester (54%), and 89% of babies are born in a health facility [15]. While full implementation of all public health innovations takes time [17], South Africa has many components in place to eliminate the vertical transmission of HIV.

However, prevention of HIV transmission and the continued well-being of the mother, child and family involve much more than HIV testing and ARV regimes. Receiving an HIV diagnosis during pregnancy often elicits feelings of anxiety, depression and social isolation [18-20]. Poor mental health and a lack of social support are, in turn, associated with decreased uptake of ARV, decreased adherence to ARV medication, and faster disease progression [21,22]. In addition to decreased social support and mental health, WLH face numerous challenges throughout the perinatal period. Women's antenatal clinic records do not follow them to the delivery centre, requiring the disclosure of HIV status to nurses in order to receive ARV medication.

Similar challenges occur at the post-birth medical appointment and the six week follow-up for infant HIV testing. Results of the baby's HIV status often take weeks or months to be available, requiring multiple maternal visits to the well-baby clinic. Co-trimoxazole is administered to 8% of infants under two months of age who are exposed to maternal HIV, but where status is not identified [23]. In addition to health concerns, a WLH must decide whether, when, and to whom she will disclose her HIV status. In particular, she must decide whether to disclose to her sexual partner, and how to encourage him to be tested for HIV.

In addition to HIV, WLH face intersecting epidemics of alcohol and malnutrition. South Africa has the highest documented rate of Fetal Alcohol Syndrome (FAS) globally [24], and the rate has been increasing [25]. Although health services should address alcohol and nutrition problems, South African antenatal programmes do not include standard screening or counselling of pregnant women regarding alcohol use. Even though vitamin supplements and folic acid are provided to enhance nutrition through antenatal care, 17% of babies are less than 2500 grams, 24% are stunted and/or malnourished under the age of 5 years old, resulting in lifelong negative health outcomes [26]. Co-morbid alcohol use and depression negatively impact infant birth weights [27] and in South African townships, post-partum depression rates exceed 30% [28]. Even low levels of maternal alcohol consumption are related to negative developmental sequelae [29]. Most South Africans consider more than one antenatal care visit unnecessary [30], therefore WLH are unlikely to receive the information, skills, and support needed to consistently maintain healthy routines for themselves or for their child.

The current project examines the effectiveness of paraprofessional community health workers, *Peer Mentors *(PM) to help WLH in the intervention to cope with a range of health and mental health challenges. Peer Mentors are WLH who have been through PMTCT and are themselves thriving, based on the theory of positive peer deviants [31]. Training of the Peer Mentors focuses on existing evidence-based HIV, child health, mental health and alcohol-related interventions [9,32-34]. *The Masihambisane Project*, which means "we walk together" in Zulu, was initiated to test the effectiveness of a Peer Mentor programme in improving the health and well-being of WLH and their babies in PMTCT services provided by the public sector [35] in a very high seroprevalence area.

## Methods/Design

### Objectives

*Masihambisane *is a cluster RCT to test the effectiveness of Peer Mentor support in addressing health, mental health, and stigma challenges faced by WLH. The challenges of chronic illnesses, mental health symptoms, infant feeding and treatment are confronted by WLH on a consistent, daily basis, therefore no single outcome can definitively validate the effectiveness of this Peer Mentor program. We focus on cumulative change across 15 indicators of maternal and infant health. The health-protective behaviors of WLH are documented over time and compared to the baseline rates. These indicators range in their uptake, for example, 92% of babies receive their vaccinations on time, however, infant HIV testing and receipt of results occurs less frequently.

We hypothesize that WLH and their infants in the intervention clinics, compared to WLH who receive standard PMTCT care, will have:

- higher maternal knowledge about HIV self-management;

- better maternal ARV adherence antenatally and at the time of birth;

- increased HIV testing of their baby at six weeks and receipt of test results;

- adherence to regular health visits and TB testing;

- infant weight of more than 2500 grams at birth with normal growth and development for 12 months;

- greater mental health, social support, and ability to cope with HIV stigma;

- higher rates of HIV testing among sexual partners; and

- higher rates of condom use with partners of serodiscordant HIV status.

In order to achieve statistical significance in the analyses, we need to recruit 1200 pregnant WLH (150 per clinic at 8 clinics) with 600 in the intervention and 600 in the standard-of-care control clinics. Enrollment criteria are: 18 years or older at the time of first assessment, less than 34 weeks pregnant at intake, HIV seropositive, and enrolled in the PMTCT programme at one of the eight study clinics. In addition, prospective participants are asked to indicate their intention to reside in the area for the duration of the study, and they must be able to give informed consent, as judged by the interviewer.

### Design

Eight rural clinics in Kwa-Zulu-Natal were matched on information collected from clinic surveys and observations regarding client load, patient characteristics, range of services offered, and rural-urban setting. The UCLA team, without first-hand experience of the clinics, then randomized clinics to the intervention or the standard care condition.

Standard care includes: dual therapy for PMTCT, referral to Highly Active Anti-Retroviral Therapy (HAART) for women with CD4 counts below 200 or WHO Stage 4 illness, infant PCR test results by six weeks of age, and co-trimoxazole for exposed infants starting at six weeks of age [36]. The intervention clinics will receive the standard care in addition to Peer Mentor support sessions. Peer Mentors were selected from the local communities, based on their HIV seropositive status and role as positive peer deviants. Peer Mentors were trained to deliver four antenatal and four postnatal small group sessions in the clinics to supplement the PMTCT program. An independent assessment team conducted in-person interviews with WLH during pregnancy, and within six weeks, six months and twelve months post-birth, evaluating each WLH and her baby's health and well-being.

### Setting

**Figure 1:**
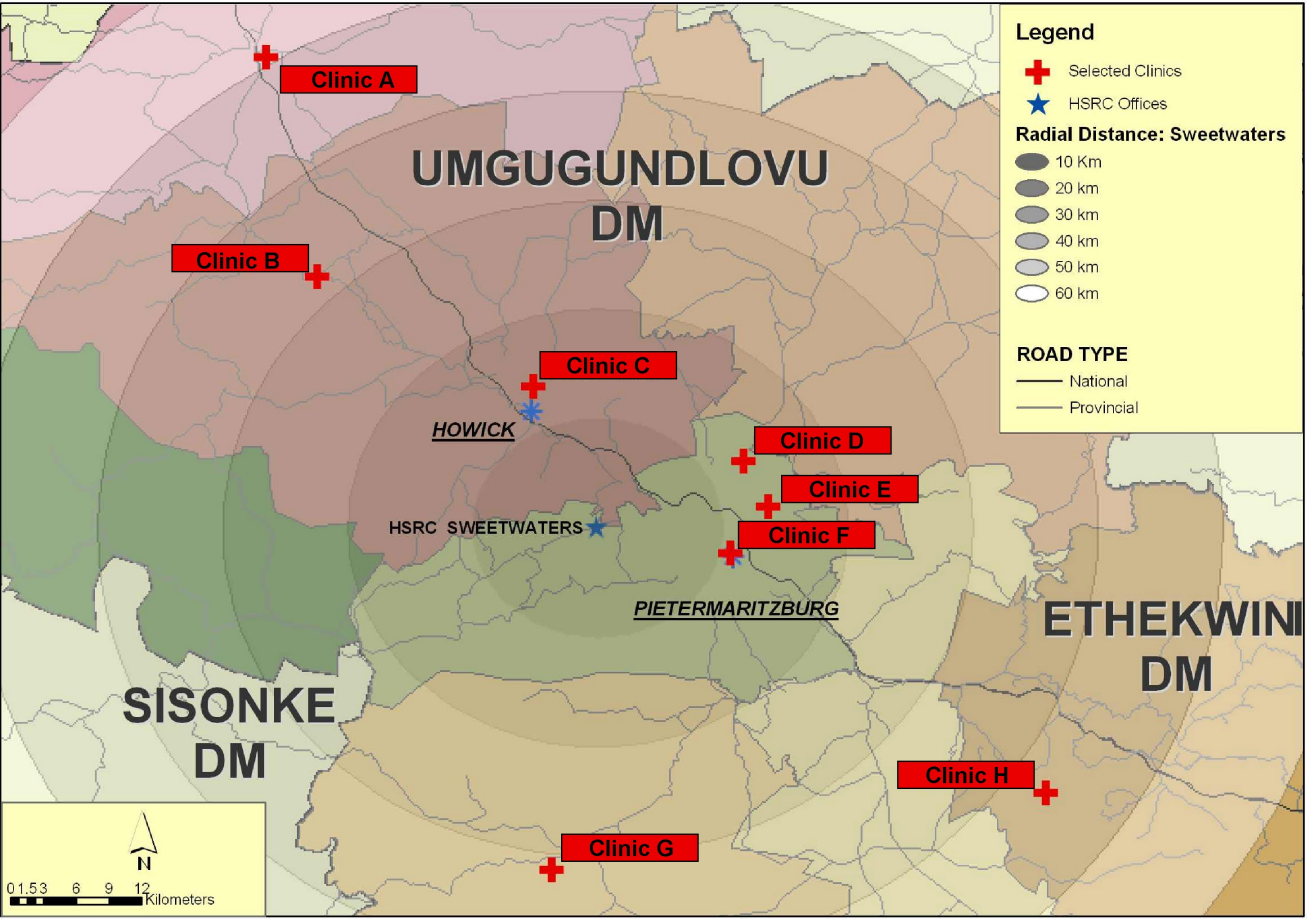
**Geographic location of Mashihambisane clinics**.

The KwaZulu-Natal (KZN) province has an HIV prevalence of 15.8% for all people over 2 years of age, the highest prevalence of HIV in the country [3]. Clinics in the *Masihambisane *project are spread over three districts in KZN (see Figure 1). Antenatal clinic data from these districts indicates that in 2007, prevalence was 40.8%, 34.1% and 41.6% [37]. Nineteen clinics that did not have on-going interventions were considered for the study sample based on proximity to main research site, type of clinic (either a community health clinic or primary health care clinic), availability of antenatal and postnatal services at the clinic, and uptake of antenatal and postnatal services (with a minimum of 300 women per annum). Table 1 lists the characteristics examined to establish the eight clinics most suitable for inclusion.

**Table 1:** Characteristics evaluated prior to clinic selection.

Characteristic	Description
**Type of Clinic**	Community Health Centre offers wide array of services 24 hours per day; Primary Health Care Clinic offers delivery of primary health care 8 am-5 pm daily; Mobile Primary Health Care Clinic offers delivery of primary health care through mobile unit 8 am-5 pm daily.

**Size of Clinic**	Determined by the type and scope of maternal and child services, including antenatal, post natal, PMTCT, and the number of first and repeat clinic attendees each month.

**Geographic Location**	Determined by logistical feasibility, availability for telecommunication network communication signal & space available for research activities.

**Risk of Contamination**	Control group is determined to be at no- or low-risk for contamination of intervention activities.

### Collaboration

The study is a collaborative effort between the Global Center for Children and Families, University of California, Los Angeles (UCLA; M.J. Rotheram-Borus, Principal Investigator) and the Child, Youth, Family and Social Development programme at the Human Sciences Research Council, South Africa (HSRC; L. Richter, Principal Investigator).

### Research Ethics and Approval

The Institutional Review Board of the University of California, Los Angeles (UCLA, G06-05-062) and the Research Ethics Committee of the Human Sciences Research Council in South Africa (HSRC, REC 4/07/03/07) have approved the study and oversee adherence to the study protocol over time. A six-member Data Safety and Monitoring Board (DSMB), consisting of local and international experts, monitors the implementation of the trial. National, provincial, district, and municipal health authorities approved the study and its protocol, and set the conditions of the standard care practice. Quarterly meetings are held with a Community Advisory Board (CAB) consisting of 25 local stakeholders who serve as liaison between research staff and the community, advising on study policies and keeping the community informed of progress. Regular feedback meetings are held with health authorities.

### Research activities and procedures

#### Pilot activities

*1. Clinic selection and matching criteria*. Geographic maps, administrative data from the health district, and a month-long observational period in each clinic provided information necessary for selection of clinics for study inclusion. Clinics were identified on the following criteria: a) location - rural (n = 4) or urban (n = 4); b) clinic type - community (n = 2) or primary health care centre (n = 6); c) size of catchment area; d) monthly census of pregnant women; and e) resources such as space, staffing, and transport.

*2. Piloting of the assessment and the intervention*. All instruments and intervention sessions were piloted through focus groups and key informant interviews. These pilot activities involved officials from the KZN Department of Health, including Cluster Heads, District Managers, Medical Managers and Programme or Clinic Managers, Department of Health Clinic Service Providers, and clinic staff members. WLH also participated in pilot sessions of the planned intervention.

*3. Programming and bench testing of mobile phones for collection of assessment interviews*. Data is collected on mobile phones running a survey software package (Mobile Researcher; http://www.populi.net/mobileresearcher/). This platform allows the phone to be used to collect and upload numeric, voice and text data. Two models of phones were used in the pilot - the Nokia E61i (a business phone similar to a PDA) and the Nokia 2630 (an inexpensive candybar phone). Initial training covered practical aspects of phone navigation, checking for software updates, use of the software, and uploading data to the central server. Staff spent two weeks familiarising themselves with the baseline survey on the phone. During this time multiple tests were run to ensure that all data entered on the phone was correctly uploaded and that the response entered on the mobile corresponded to the value stored in the database.

*4. Standardization of clinic procedures and training updates consistent with national guidelines*. During clinic observations, processes for antenatal registration, routine HIV testing, post-test counselling, and antenatal care were identified. The team, health administrators, and clinic staff spent two months collaboratively standardizing and training clinic staff in these procedures. The re-training focused on ensuring that all personnel were equipped to deliver standardised and routine PMTCT antenatal and postnatal health care, as prescribed by current departmental policies and guidelines. Strategies to increase HIV Voluntary Counselling and Testing (VCT) service delivery and uptake were also implemented in all clinics.

### Study Procedures

#### Recruitment

A protocol was written for each clinic to ensure that recruitment was standardized across clinics despite differences in procedures. The intake nurse in the antenatal clinic services introduces the *Masihambisane *research assistant to pregnant women in the waiting room, and the women are provided with a brief overview of the study and key PMTCT messages.

HIV VCT is conducted during the first antenatal visit using an opt-out procedure. During counselling by a clinic nurse, all women, regardless of HIV status, are offered the opportunity to speak to *Masihambisane *staff. *Masihambisane *is branded as a program to support pregnant women, not specifically WLH. The *Masihambisane *staff member reinforces key PMTCT messages tailored to each woman's HIV status. In intervention clinics, the staff member also offers all women, HIV-positive and HIV-negative, a referral to speak with a Peer Mentor for additional support regarding their pregnancy, health or HIV status.

In the case of WLH, the Peer Mentor discloses her own serostatus, empathizes with the WLH and offers follow-up for enrollment into the research study. The WLH is given material about the study and the consent form to read carefully at home. At a second enrollment meeting, the research assistant reads aloud a script that describes the research study, probing for the WLH's understanding of key concepts. Women are reassured that her decision to enroll will not affect services received at the clinic. With voluntary informed consent, a baseline interview is conducted using the mobile phone to collect and record data. In the intervention clinics, the WLH is then referred to the Peer Mentor for enrollment into the small group support programme.

#### *Masihambisane *Intervention

The Peer Mentors work full-time in the intervention clinics and are integrated into the Department of Health PMTCT programme at each site. WLH are invited to attend a series of four antenatal and four postnatal sessions, scheduled to coincide with their routine antenatal and well-baby clinic visits. Sessions last 60 to 90 minutes, and are attended by four to ten WLH who meet to discuss, roleplay, share, and practice strategies to address issues critical to effective PMTCT. Each session proceeds in a similar sequence: WLH arrive, sing and pray together, and share successes and joys from their week. The Peer Mentor shares her triumphs and challenges as a WLH and acknowledges the importance of the support group for her. A topic is introduced for the day's meeting and a brief demonstration of the content message is presented, then the group brainstorms the types of challenges the WLH will encounter if she tries to implement this knowledge in her life. Finally, goals are set for the upcoming week and a song and a prayer end the meeting.

Each of the following issues is covered by the intervention:

1) *Living Positively*. Using a jar with different coloured jelly beans, the Peer Mentor shows that all families in KwaZulu-Natal are affected by HIV and the WLH is not alone, in particular she has support in the group leader and group members. WLH are encouraged to attend all clinic appointments. Women are provided with referral letters to the couple to encourage them to get tested together for STIs. Women discuss to whom they want to disclose their serostatus and roleplay the process of disclosure and asking for emotional support.

2) *Keeping Myself and My Baby Healthy*. The Peer Mentor helps WLH understand why they must avoid smoking and drinking, eat healthily, take vitamins and medicine as prescribed in antenatal care, get regular exercise, seek and maintain social support, and do things they enjoy. A realistic black baby with Fetal Alcohol Syndrome is presented and the characteristics and life-long consequences of alcohol on babies are discussed. A raw egg is broken in a cup of alcohol and women watch as the egg poaches at room temperature to illustrate adverse effects of alcohol on brain development *in utero*.

3) *Being Prepared*. The PM and WLH discuss the importance of taking medicines, keeping clinic appointments, registering her child to receive the child support grant (models provided and strategies for problem solving challenges to obtaining ID documentation are reviewed), and keeping track of health records. Women are provided a card to give their delivery nurse that says, "I am HIV+, please make sure that I get ART" and women discuss the importance of disclosing status. Additionally, the Peer Mentor demonstrates ART wrapping and appropriate administration method.

4) *Choosing an Exclusive Feeding Method*. WLH are urged to use only one feeding method exclusively for six months, formula feeding is discouraged unless the WLH has clean water on home premises, a toilet, and enough money to buy the product on an ongoing basis. However, clinics do distribute milk powder to all WLH at childbirth. The challenges of using only one feeding method for six months are brainstormed: WLH identify the different types of concerns, such as too little food, ritualistic healing substances, advice from different persons (partner, mother-in-law, neighbors), getting support for child care, and building a strong bond.

5) *Living a Long Life Together*: The steps in obtaining a child support grant are outlined, examples of concrete ways to seek an identity book are provided, the Road to Health card is shown with dates for baby immunizations, healthy meals are presented, pictures of WLH prior to and following ARV (Lazarus effect) are shared, the schedule for getting regular maternal health checkups, including TB tests are outlined, the importance of the six-week HIV test for the baby and returning for results are emphasised, and strategies are reviewed to avoid infecting a partner with HIV.

7) *Being Partners and Parents*. This session stresses getting your partner tested, deciding whether to have another baby, using condoms, dealing with a partner's alcohol use and any multiple partnerships he might have, and finding support for domestic violence.

8) *Enjoying Life*. PM and WLH discuss the importance of taking time for oneself, engaging in pleasant activities for oneself and the baby, maintaining close friendships and other relationships, and asking for and accepting help from others.

Three tools are used in all intervention sessions. To increase awareness and skills for emotional regulation, WLH are taught to use *Feeling Cups *to calibrate their feelings. A clear glass is filled with different levels of water, depending on their degree of uncomfortable (full) feelings. *Tokens *are exchanged between members. These are small chips that indicate positive feelings towards one or more group members. Finally, WLH are provided with an *empty jar and beans *to count the number of times they adhere to their goals.

We have developed a variety of materials, manuals, and videos on PMTCT. Workbooks for pregnant women were adapted for HIV, alcohol and malnutrition content from existing evidence-based interventions [8]. All materials are in Zulu, after being translated and back-translated into English to ensure their accuracy and appropriateness. The colours and images were selected by the intervention team, the Peer Mentors, and the Community Advisory Board. In designing these learning aids, we followed the process for developing materials in Tanzania [38] where "job aids" were developed that are culturally tailored, easy to use, and feasible to distribute. All materials were used in the pilot interventions and we debriefed pregnant WLH, nurses in health clinics, and supervising Peer Mentors in development of the materials to ensure cultural appropriateness.

No incentive is given for participating in the intervention sessions, but participants receive tea and biscuits on arrival, and R30 (about $3.50) towards their transportation costs. Several sessions are held per day at the clinic and are rotated, to enable women to attend any session on the same day as their routine health care appointments. Attendance data is collected at all sessions.

Quality control is managed at three levels: 1) within the clinic by the site coordinator; 2) across clinics by the centralized Intervention Coordinator; and 3) monthly and quarterly by international and domestic intervention specialists. A checklist is completed by the Intervention Coordinator when observing Peer Mentors' groups and provided during routine supervision to Peer Mentors.

### Data Collection

**Figure 2:**
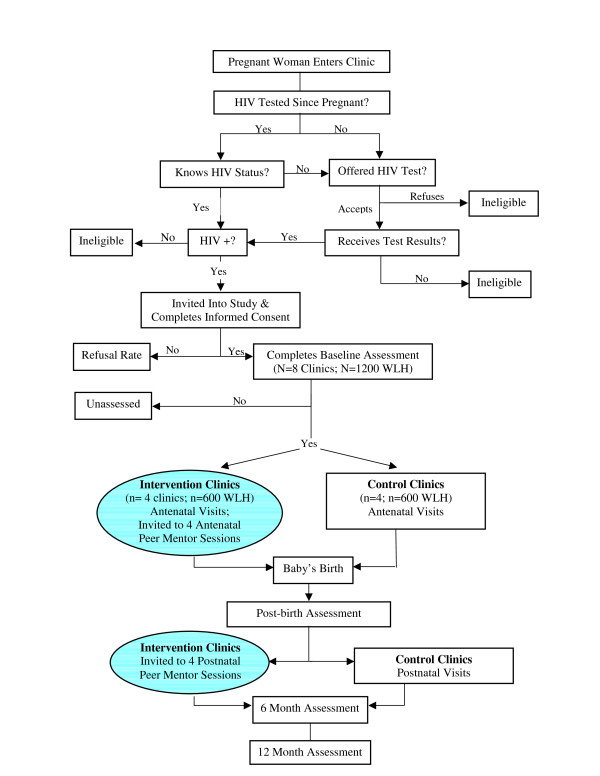
**Participant flow through RCT**.

Four types of data are consistently monitored:

*1. Clinic data to ensure matching of intervention and standard care clinics. Masihambisane *staff collect aggregated clinic level data for all study clinics: total patients, women attending antenatal care, HIV tests, results received, and participants enrolled in the study. This allows monitoring of enrollment, attrition, and those receiving PMTCT. Figure 2 shows the flow of participants through the data collection process from entry at the clinic, through the consent process, session attendance and post birth assessment.

*2. Medical record reviews. Masihambisane *staff collect and review the medical records of each participant and extract routine health data including: antenatal weight and height, blood pressure, HIV test results, CD4 count, rhesus factor, syphilis and other sexually transmitted infection test results, haemoglobin/anaemia, tuberculosis, preventative medications used such as AZT and NVP, date of delivery, baby weight and length, birth complications, vitamins dispensed, mother's TB and HIV test results, PCR test results, and immunizations.

*3. In-person interview assessments*. A team of two interviewers is placed at each clinic on a full-time basis to interview WLH after their first antenatal visit and six weeks, six months, and twelve months post-birth. All assessments are confidential, and are administered by a trained research assistant, supported by a computerized mobile phone interview protocol. Mobile phones are less expensive than laptops or PDAs, less likely to be stolen, more user-friendly for research assistants and Peer Mentors, and easily provide management information on the duration and location of assessments, enabling data collection with integrity. The program improves data quality by performing simple logic and range validation as data is entered. Skip patterns are also automated, and sections of the survey can be automatically repeated; for example, to cover the same questions about multiple children or partners. Most important, the phones retain all data when no signal is available and automatically upload data when in range of a signal. Uploads occur approximately every 60 seconds, when network coverage permits. The application is pre-configured with identification information, preventing data submission by unauthorised users. In addition, a compromised handset can be blocked at any time to prevent both data uploads and downloads. Secure Sockets Layering (SSL) is used to ensure that all data transferred between the device and the server is encrypted. Uploaded data is available for review, management and export via the web-based *Masihambisane *data platform. Only senior investigators are able to access the data platform. The servers hosting the data are located in Johannesburg, South Africa, and offer full and redundant data protection and security.

Each of the assessments take between 60 and 90 minutes to complete, and cover family background, health, sexual partnerships and behaviours, disclosure, knowledge about HIV issues, daily routines, alcohol and drug use, general knowledge about child care and infant feeding plans, as well as all process and outcome data.

*4. Quality of the intervention delivery*. All intervention facilitators are trained for two months prior to beginning the project. The training includes review of the intervention manual, provision of prompts and materials to support all session materials, and practice role plays with WLH. Supervision is conducted by supervisors who rotate to each site every two weeks to observe and provide feedback, and supervise weekly group sessions. Senior collaborators provide training on a quarterly basis and supervisors give support to Peer Mentors to help them cope with their own feelings about the difficult situations in women's lives. All sessions are conducted by co-leaders to ensure that all procedures are followed as outlined in the manual.

### Data Analysis

WLH are assessed at six days, six months, and twelve months post-birth. Outcomes are compared to health care data routinely collected for mother and child.

The primary outcome is a composite score calculated as the sum of indices for the presence (1) or absence (0) of maternal and child health and well-being. Indices encompass five domains. 1) ***Child health status *** includes birth weight and length being within one standard deviation of World Health Organization (WHO) standards, normal development according to WHO norms for motor milestones, absence of FAS symptoms, and positive parenting as assessed by the Parenting Stress Index short form [39] and a bonding questionnaire [40]. 2) ***Healthcare and health monitoring *** include the number of antenatal, postnatal, and HIV-related clinic visits. Monitoring includes understanding CD4 count and adherence to all prescribed anti-retrovirals. 3) ***HIV transmission-related behaviors *** include disclosure of HIV test results to partners, requesting partners to test, consistent use of condoms, use of single feeding method until six months, baby testing at six weeks, baby prescribed and administered antibiotics at six weeks, maternal AZT during pregnancy, infant NVP at birth and AZT after birth, and disclosure of baby's status to partner or family. 4) ***Mental health *** includes the Edinburgh Postnatal Depression Scale [40] and the General Health Questionnaire. 5) ***Social support *** includes an adapted scale from Barrera [41] assessing instrumental, emotional, and childrearing support as well as family, neighbour, and community support for healthy acts. The indices are monitored throughout the intervention and at the final assessment.

Intervention effectiveness is assessed by comparing the composite outcome over 12 months between the intervention and standard-of-care control conditions. These composite scores will be compared using multilevel models, i.e., hierarchical models and random effect models, which account for clustering of repeated assessments within individuals and clustering of individuals within clinics [42]. Multilevel models can be applied to normally distributed and non-normally distributed outcomes, we will choose the most appropriate model based on the data. If data transformations sufficiently normalize the composite outcome, e.g., a logarithmic transformation to reduce skewness, we will fit a linear model. If a normal assumption is not appropriate, we will categorize the data and use a longitudinal logistic regression for binary responses [43] and longitudinal ordinal logistic regression to look for shifts in ordinal response levels between the intervention conditions [44]. In all analyses, clustering within clinic will be taken into account, and multiple imputation methods will be used for missing individual outcome scores [45,46].

While the primary outcome composite score will provide a summary of the overall effectiveness of the intervention program, we will also examine each outcome component for a more detailed interpretation. This will be done using longitudinal logistic regression for binary data, to determine whether, and in what direction, the intervention is associated with changes in each component.

Finally, dose-response analysis will be conducted to test for relationships between the number of intervention sessions attended, as well as time of recruitment, and the composite score. The initial patterns of the outcome measures will be examined through descriptive summaries. If necessary, transformations will be conducted to approximate normality, homogeneous variance and linearity in the model.

## Discussion

The use of inexpensive and widely available mobile handsets, mobile survey software and existing cellular networks provide an electronic data collection platform that is a particularly innovative aspect of this study. This is especially valuable given the low resource setting in which the research is being conducted - many of the study clinics do not have desktop computers, landline telephones or easy vehicle access. Despite these challenging circumstances, the mobile data collection platform has made many of the established benefits of electronic data collection available. Two examples highlight this point. First, data quality has been aided though a system of automatic checks on the data that are performed overnight. One such check ensures that at the end of the day all research assistants have submitted the required data. If data is not submitted, or the submitted data fails predefined error checks, an error report is generated and made immediately available to team leaders who follow up with the field team in the clinic. Second, a report of distressed feelings during an assessment can be quickly addressed through cell phone communication. This is achieved through a database trigger which is set to automatically run a script every time a new assessment is submitted. Once triggered, the script scans the mental health data supplied by the participant. If the participant mentions having thoughts of self harm, email and text message (SMS) communications can be dispatched immediately to the counselling psychologist on the team for follow up.

Transmission of the HIV virus between mother and child is preventable; PMTCT provides a technology that can dramatically reduce new infections. Unfortunately a number of socioeconomic and health system challenges reduce the efficacy of PMTCT in South Africa. Observations during the first year of this trial indicate that poverty, cultural norms, inability to disclose one's status and inaccurate knowledge about dual therapy detract from standard PMTCT. At a systems level there are numerous challenges faced by health providers in offering a viable and effective PMTCT program. Inconsistent availability of Voluntary Counselling and Testing (VCT) counsellors and nursing staff at the clinic, understaffing, deviation from the PMTCT protocol guidelines as laid out by the National Department of Health, inflexible labour practices, slow turnaround times for CD4 and PCR results, and ARV drug shortages hamper the efforts of primary health care providers.

As described previously, even with perfect implementation, a solely pharmacological delivery approach to PMTCT is unlikely to be sufficient. This is particularly true given that in South Africa many WLH live in challenging circumstances. High levels of poverty, alcohol use and abuse, and domestic violence interact with HIV-related stigma and discrimination to present WLH with many challenges. This project will provide valuable new data on the effectiveness of an intervention that focuses on providing WLH with Peer Mentor support in facing the myriad daily challenges related to maternal and infant health and well-being.

## Competing interests

The authors declare that they have no competing interests.

## Authors' contributions

MJR contributed to study conception, design and critically revising the manuscript. LMR contributed to study conception, design, drafting and critically revising the manuscript. HVR contributed to study design, drafting and critically revising the manuscript. AVH, NM, LMk, LN and LMt contributed to the acquisition of data, drafting and critically revising the manuscript. AS, TR and MT contributed to study design, drafting and critically revising the manuscript. JDK contributed to drafting and critically revising the manuscript. SC was responsible for statistical data analysis, drafting the results section, and editing the manuscript. All authors read and approved the final manuscript.
